# Health Topics on Facebook Groups: Content Analysis of Posts in Multiple Sclerosis Communities

**DOI:** 10.2196/10146

**Published:** 2019-02-11

**Authors:** Sara Della Rosa, Falguni Sen

**Affiliations:** 1 Global Healthcare Innovation Management Center Department of Management Systems Fordham University New York City, NY United States

**Keywords:** social network, health information, health care internet, content analysis, Facebook

## Abstract

**Background:**

Social network sites (SNSs) are being increasingly used to exchange health information between patients and practitioners, pharmaceutical companies, and research centers. Research contributions have explored the contents of such exchanges discussed online. They have categorized the topics discussed and explored the engagement levels of these discussions.

**Objective:**

This research aimed at investigating the potential role of SNSs in health care. Specifically it provides an information-clustering analysis of the health information available on SNSs and develops a research design that allows an investigation of this information in enhancing health care research and delivery. In addition, this research aims at testing whether SNSs are valid tools for sharing drug-related information by patients.

**Methods:**

This research is based on a specific chronic disease: multiple sclerosis. We searched Facebook to identify and research the social media groups related to this condition. The analysis was restricted to public groups for privacy concerns. We created a database by downloading posts from two main groups (in the English language). Subsequently, we performed a content analysis and statistical analysis; this allowed us to explore the differences between categories, their engagement levels, and the types of posts shared. The mean level of engagement for each topic was analyzed using a 1-way analysis of variance.

**Results:**

From a sample of 7029 posts, initial results showed that there were 8 information categories that resonated (percentage of times the topic appears in our sample) with those who post on Facebook: information and awareness (4923/7029, 70.04%), event advertising and petitions (365/7029, 5.19%), fundraising (354/7029, 5.04%), patient support (217/7029, 3.09%), drug discussion (144/7029, 2.05%), clinical trials and research studies (59/7029, 0.84%), product and drug advertising (48/7029, 0.68%), and other (919/7029, 13.07%). Initial analysis showed that comments and likes (as measures of engagement level) are the most frequent indicators and measures of level of engagement. Our results show a high engagement level (in terms of views, likes, comments, etc) for patient support and information and awareness. In addition, although drug discussion had a low resonance, it had an unexpected highly engagement level which we found worthy of further exploration.

**Conclusions:**

SNSs have become important tools for patients and health care practitioners to share or seek information. We identified the type of information shared and how the public reacted to it. Our research confirmed that the topics discussed in social media related to specific diseases such as multiple sclerosis are similar to the information categories observed by other researchers. We unexpectedly found other categories such as drug discussion. These and other results of our study enhance our understanding of how content is disseminated and perceived within a specific disease-based community. We concluded that this information has useful implications in the design of prevention campaigns, educational programs, and chronic disease management.

## Introduction

Social network sites (SNSs) are “Web-based services that allow individuals to (1) construct a public or semipublic profile within a bounded system, (2) articulate a list of other users with whom they share a connection, and (3) view and traverse their list of connections and those made by others within the system” [1]. In health care, social networks have been adopted relatively late with respect to other industries due to privacy, reliability, and ethical issues. However, there has been a huge increase in the number of information and communication technologies applied within this sector leading to an increased use of social networks. As a result, researchers have focused their attention either on a specific online service such as health social networks (eg, PatientsLikeMe) or on the use of existing SNSs (eg, Facebook) for the purpose of researching health care discussions. Although there is some published research on the use of SNSs in health care demonstrating positive contributions, studies are mostly focused on the usefulness of SNSs in clinical trial recruitment and patient-initiated studies [2-4] *.* In addition, some papers study patient interactions within online health communities in terms of the effect of the data these interactions generate in advancing health care by creating higher levels of efficiency in the delivery of care and more effective patient-centric outcomes [5-7] *.*

Facebook is a popular SNS used in the study of health care–related discussions. Bender et al [8] investigated the purpose and use of Facebook groups related to breast cancer. Their research was based on the result of a content analysis of the statements of each group (eg, title of group, description of group, information in the Recent News section, discussion posts, and wall posts). They found that on Facebook there were about 620 breast cancer groups and they were created for fundraising (44.7%), awareness (38.1%), product or service promotion related to fundraising or awareness (9%), and patient/caregiver support (7%).

Setoyama et al [9] studied the participation in online communities by patients affected by breast cancer and the related benefits thereof. They found that there is a difference between posters and lurkers and that posters felt they received more benefits from online communities than lurkers did. The benefits perceived included emotional support, helping other patients, and an opportunity to express their emotions. Researchers found that even lurkers gained a certain amount of peer support especially related to getting advice and insight/universality.

Moorhead et al [10] reviewed the literature to identify the uses, benefits, and limitations of social media for health communication. They found that 6 key benefits characterize SNSs: (1) increased interactions with others; (2) more available, shared, and tailored information; (3) increased accessibility and widening access to health information; (4) peer, social, and emotional support; (5) public health surveillance; and (6) potential to influence health policy. They also identified limitation such as quality concerns and lack of reliability, confidentiality, and privacy. Given the growth of SNSs, studies have now tried to analyze how the information available is actually structured in terms of content and topics discussed.

Hale et al [11] searched the top 20 health conditions on Google and selected the Facebook pages that corresponded to them. In doing so, they compiled a list of the top 50 Facebook pages for each health condition and analyzed them. They employed a content analysis technique on 522 pages and found that the most common type of page was marketing and promotion (32.2%) followed by information and awareness (20.7%), Wikipedia-type pages (15.5%), patient support (9.4%), and general support (3.6%). In that paper, they showed how the type of page was different for different health conditions.

Studies from Bender et al [8] and Hale et al [11] focus on content analysis of the information that is publicly available at the groups/pages level (eg, group purpose statement) but not at the posts level (all posts shared by individual users). Our study, on the other hand, develops a method to categorize online content from actual posts. This allows us to simplify the large number of members posting on the sites selected (24,915 members participating).

There are a number of challenges to doing research using content analysis of information in SNSs. The first one is getting the data from an SNS. This requires access to the data and an application program interface (API) that allows the researcher to collect the data. The second challenge is to develop an algorithm that is able to detect the type of post. This is a complex task requiring a deep understanding of the words used by patients in their posts. The researcher needs to be able to recognize how single words are used in every type of post in order to develop the algorithm. In this study, we aimed at developing a framework for categorizing online content in clusters of related terms. This allowed us to interpret the huge amounts of data available online and make them usable for further analysis. Clustering information has always been a valuable practice and, with the increasing use of the internet, it becomes even more important because it provides a practical tool for using information publicly available online [12]. This clustering will enable an analysis of public reaction to types of information shared online and provide a more complete understanding of the usefulness of SNS-generated data for policy and management purposes.

There is little investigation in the literature of online drug discussions. There is probably an assumption that patients do not discuss use of drugs due to privacy concerns. It also appears that the methodology used by other researchers in gleaning content from posts does not allow for the investigation of drug-related discussions. This study explores the existence of drug-related discussions in public Facebook posts by patients. We further explore if the posts on specific drug use in a disease-focused SNS receive the same level of attention given to other categories such as awareness and marketing. We feel that this is indicative of how transparency and accurate information by gatekeepers may be important in proper adherence to therapy by patients. It may also suggest the need for pharmaceutical companies to create more interactive sites providing more transparent information and quicker response to drug-related issues of concern to patients. This patient feedback could also give useful information on the pros and cons and effectiveness of some therapies.

In this paper, we looked at the following research questions:

- Do our results using multiple sclerosis (MS) public posts confirm, disconfirm, or integrate the results of Bender et al [8] and Hale et al [11] in terms of categories of posts?
- Do patients share information on medication in public posts? If so, what is the nature of these posts? Is the attention received by such posts similar to the attention received for other types of posts?

## Methods

### Data Collection

This work is based on an SNS dataset of MS groups on Facebook that were investigated. MS is a neurological condition that affects the central nervous system. Patients with this condition have very active online communities, and this allows for good data availability. The dataset is composed of posts collected from 2 different MS groups for which the data were available: Multiple Sclerosis Foundation, with 16,376 members, and Multiple Sclerosis Trust, with 8539 members, for a total of 24,915 members. Both groups are public. We downloaded all available posts from both groups (4000 posts from the first group and 7900 from the second group). The next step involved cleaning the posted data by removing those shared by administrators of the groups and blank posts (where only links were shared). This resulted in 1071 posts from the first group and 5958 from the second group for a total of 7029 posts. We downloaded the data using Facebook’s Graph API available online, which is developed using Python. This program allowed us to get the posts’ message and other variables including those described in Table 1. The data collection was completed from April to May 2017 and included posts dating from October 11, 2011, to May 16, 2017. The data analysis was performed from May to August 2017.

We developed a qualitative content analytic model in-house using the approximately 1000 posts from the first group (also referred to as the small dataset). We then applied this model to the full dataset.

The model was developed in the following way:

- Step 1: We manually tagged the small dataset looking for 8 categories of topic, some of them selected from the literature and some of them built by us while reading the posts and realizing the presence of certain recurring topics in the dataset.
- Step 2: We identified those words that were more frequent for each category. For example, the drug discussion posts had a unique characteristic of having the unigram mg (for milligrams), name of an MS drug, pills, etc. All of the categories have unique characteristics, and we used these to identify the category and teach the code how to recognize a certain post.
- Step 3: We assigned high weights to the words that were obvious indicators of a certain category. We assigned these weights based on relatedness of the word to that category. For example, it is clear that the use of the word Copaxone is related to drug discussion. Thus, we would give Copaxone a high weight for the category drug discussion. Similarly, use of mg in a discussion also indicates inclusion in the category drug discussion. This system of weighting obvious words helps control for ambiguity in classification of posts.
- Step 4: We ran the code on the full dataset.

### Data Analysis

Table 2 provides the description of each category of post. As mentioned in the earlier section, the descriptions were derived from previous literature or created by us and represent the synonyms and related terms captured by our coding algorithm.

**Table 1:** Variables available in the dataset and their brief description.

Variable	Description
status_id	Status message ID
status_message	Status message in the post
link_name	Name associated with the link shared in the post
status_type	Description of the type of a status update
status_link	Unique link that allows post to be retrieved
status_published	Date and time the post has been shared
num_reactions	Sum of total reactions (likes, loves, wows, hahas, sads, angrys)
num_comments	Count of comments the post received
num_shares	Count of how many times a post has been shared
num_likes	Count of the like reactions the post has received
num_loves	Count of the love reactions the post has received
num_wows	Count of the wow reactions the post has received
num_hahas	Count of the haha reactions the post has received
num_sads	Count of the sad reactions the post has received
num_angrys	Count of the angry reactions the post has received

**Table 2:** Content shared on multiple sclerosis groups in our sample by cluster.

Category	Description
Clinical trials and research studies (new category)	Advertising or sharing experience of participation in clinical trials, research studies, and testing medical devices.
Drug discussion (new category)	Asking for or giving information about drugs and treatments. Patients usually ask for other patients’ experiences or suggestions associated with a specific drug. Only posts that included a specific drug name were included in this category.
Event advertising and petitions	Advertising an event’s purpose, time and location. Petitions are usually addressed to the government as requests for actions.
Fundraising	Post asking for money donations created to attract financial resources for multiple sclerosis events, products, services, etc.
Information and awareness	Patients talk about their experience living with the condition in order to receive impressions from others or users that aim at rising awareness by sharing research pages, recommendations, etc.
Patient support	Emotional and informational support for patients. This type of content helps to improve their sense of the condition and to accept it and may include motivational messages from others.
Product and drug advertising	Advertising from pharmaceutical companies (or related) to promote drugs, products, services, treatments, devices, etc.
Other	Posts that don’t belong to any of the described categories.

Regarding the first category, clinical trials usually involve the participation of human volunteers who receive an intervention, while research studies, in this specific setting, require less invasive participation that may include, for example, participation in a survey for medical or pharmaceutical purposes.

We used R (R Foundation for Statistical Analysis) software for data analysis, and we used nonparametric analysis of variance (ANOVA) to answer our research questions. Frequency counts and box plots provided a description of the importance of categories. We tested whether difference in the level of engagement is statistically significant in different categories using a 1-way ANOVA. We tested normality with a Shapiro test (only on the first 5000 observations due to this test limitation in R), histograms, and boxplots. Our data are not normally distributed, and we addressed this by using a nonparametric 1-way ANOVA.

## Results

### General Findings

On a sample of 7029 posts, initial results show that there are 8 categories of topics discussed in the posts related to multiple sclerosis on Facebook. Table 3 provides the percentage of posts for each category in our sample.

Information and awareness was the most discussed topic, followed by event advertising and petitions, fundraising, and patient support. Three clusters that are directly related to pharma-centered topics are at the bottom of this ranking: drug discussion, clinical trials and research studies, and product and drug advertising. Some of the posts could not be located in any of the topics and were classified as other.

### Most Shared Topic

As mentioned before, the information and awareness content shared in the analyzed MS groups is dominant. Investigating this category further, we wanted to understand if there were any additional subcategories that could be detected in this topic such as people who share personal health stories or people who talk about others’ experiences. We found that, in fact, posts related to information sharing and awareness about MS are shared by patients themselves, a third person (including parents, relatives, friends, etc), and those unrelated to any person. We developed an algorithm that allowed us to detect the 3 subcategories based on the unigrams and bigrams (a contiguous sequence of n items from a given sequence of text, respectively size 1 and size 2) that were selected in accordance to the definition of the searched subcategories. Words included in the search for the first person category were those associated with sharing a personal story, such as “I am” and “I experienced” while words associated with the third person category were those who indicated a post generated by someone talking about a friend or a relative and include the words “she is,” “he is,” etc. All posts that were not detected as first or third person but had words such as awareness, aware, etc, and so were part of the information and awareness category were included in the general subcategory without first/third person attribution; 236 posts were not assigned to any of the 3 subcategories detected. We found that 41.39% (1940/4687) of the posts available in this category belong to the first person subcategory meaning that more than 1/3 of the content shared in this category was generated by the patients themselves. A total of 7.68% (360/4687) of the posts were generated by people who had a friend or relative affected by MS. More than half of the posts in this category (2387/4687, 50.93%) were general posts that shared information with no particular references to a person.

### Pharma-Centered Topics

The data show that clusters directly related to pharma-centered topics (drug discussion, clinical trials and research studies, and product and drug advertising) represent only a small portion of the posts (251/7029, 3.57%). It is also possible that privacy concerns dissuaded participants from sharing information.

**Table 3:** Topics discussed and frequency of posts (N=7029).

Category	Value, n (%)
Clinical trials and research studies	59 (0.84)
Drug discussion	144 (2.05)
Event advertising and petitions	365 (5.19)
Fundraising	354 (5.04)
Information and awareness	4923 (70.04)
Patient support	217 (3.09)
Product and drug advertising	48 (0.68)
Other	919 (13.07)

However, the fact that there was indeed social network discussion on pharmaceutical-related issues was unexpected due to privacy concerns and may provide us with useful information.

**Figure 1:**
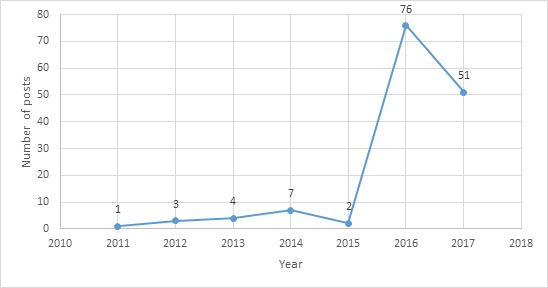
Drug discussion posts timeline.

Within this category, the high percentage of drug discussion was intriguing. The timeline in Figure 1 shows how discussion in this area has grown in the last few years. Looking through the posts, we find that this discussion centers on the effectiveness of other patient’s therapies, side effects, contraindications, natural therapeutic alternatives, and potential new drugs. If the popularity of these posts grows, we may find useful information guiding the treatment and development of new drugs.

We believe that the drug discussion category is worthy of further investigation because this is where patients share detailed information about their treatments and their related experience. The analysis indicates that a spike was observed starting from 2015—35.4% (51/144) of the drug discussion posts were published in 2017, 52.8% (76/144) in 2016, 1.4% (2/144) in 2015, 67.4% (97/144) in 2014, 2.8% (4/144) in 2013, 2.1% (3/144) in 2012, and 0.7% (1/144) in 2011. No further information was available to investigate the reasons. This phenomenon may be due to the fact that Facebook groups have marketing campaigns to increase the participation or incentivize the participation in the social group by changing the group policies. While we have no systematic evidence, it may be possible that endogenous factors such as a price reduction due to patent expiration may have increased the availability and use of MS drugs resulting in greater discussion.

We developed an algorithm that allowed us to detect 5 subcategories based on the unigrams and bigrams selected in accordance to the definition of the searched subcategories. Words included in the search for the request drug information category were those associated with words such as “does anyone,” “experienced,” etc. Words associated with the side effect category were those such as side effect, problem, hate, etc. In the new treatment category, we looked for words such as “approved,” “FDA,” etc. For the alternative medicine category, we detected words such as “marijuana.” In a total of 144 posts, 138 posts related to drug discussion were clustered in subcategories and 6 posts were not assigned to any subcategories, hence not included in the analysis.

We find that patients frequently (62/138, 44.9%) ask their peers for information on drugs.

I seen my doctor today and wants me to try the new once a month shot called zinbryta does anyone take this.

Patient

Patients also have a high level of information-sharing related to their side effects (48/138, 34.8%).

Hi all Is anyone here on Zinbryta (daclizumab)? Previously was on Gilenya but had to come off due to really low blood count and skin cancer as side effects. Was on tecfidera but hate it so now going to try this as a once a month injection. Feedback appreciated.

Patient

Patients also showed a certain propensity to discuss topics related to new treatments (15/138, 10.9%).

I've been searching to find out if Ocrelizumab has been approved by the FDA today, as scheduled. Can't find anything about it. Has anyone else heard anything? I just checked the FDA.gov site and they don't yet list any drugs approved for today.

Patient

Alternative medication posts have been found in this study shared by Facebook users that actively participate in the analyzed groups (11/138, 8.0%).

Medical Marijuana in the Form of Controlled-dose Capsules Now Available in New York.

Facebook group participant

Patients discuss medical contraindications in relation to certain drugs (2/138, 1.4%).

Diagnosed May 2012. I was on Copaxone for 1 year and I relapsed every 6 to 8 weeks for a year. I stopped it and have been on Tecfidera just shy of 3 years. I have decided to pull the plug on Tecfidera due to the fact I am JCV+ and at 1200 right now, from 2400 3 months ago. WHAT medication should I consider? I'm so lost on what to take now. I want to take something and have always been aggressive with my treatment, but I feel lost now that I am JCV+ and stopping Tecifdera. HELP!

Patient

**Table 4:** Main statistics describing topics and engagement level variables for the whole dataset.

Variable	N	Mean (SD)	Median	Minimum	Maximum
Reactions	124,726	17.7 (44.1)	4	0	965
Comments	54,371	7.7 (21.7)	2	0	624
Shares	12,139	1.7 (14.2)	0	0	593
Like reactions	101,987	14.5 (36.4)	3	0	793
Love reactions	9000	1.3 (5.0)	0	0	144
Wow reactions	1627	0.2 (1.6)	0	0	69
Haha reactions	2228	0.3 (2.3)	0	0	71
Sad reactions	8702	1.2 (8.6)	0	0	452
Angry reactions	1170	0.17 (2.6)	0	0	192

### Main Statistics for Our Sample

In Table 4, the main statistics for all variables available in our dataset are shown.

From the main statistics, it appears that number of likes and number of comments have the highest engagement values in the available dataset. The number of shares and the single reactions love, wow, haha, sad, and angry have lower values in mean and median. The number of reactions variable counts all the online activity variables and reflects the positive values found in all other variables.

## Discussion

### Principal Findings

**Figure 2:**
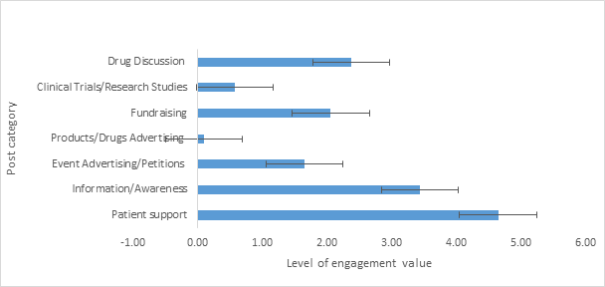
Engagement level measured as the average of 8 online activity variables (comment, share, like, love, wow, haha, sad, and angry) by the 7 topics detected in our analysis with standard error.

**Figure 3:**
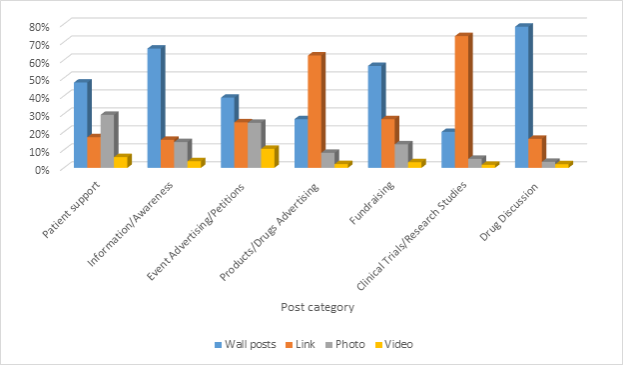
Type of post shared (wall post, link, photo, and video) by category in percentage.

In this work, we wanted to offer a deeper interpretation of the engagement level by examining its level for each category that we found. The results in Figure 2 show that there are high engagement levels for certain categories (more than 2 points in engagement level value) such as patient support (4.64), information and awareness (3.43), drug discussion (2.37), and fundraising (2.06). On the other hand, there are low engagement levels in other categories (less than 2 points in engagement level value) such as event advertising and petitions (1.65) and very low engagement levels in clinical trials and research studies (0.58) and product and drug advertising (0.10). Furthermore, we tried to understand the differences in terms of types of post used for each category, and in Figure 3 we offer a visualization of the use of wall post, link, photo, and video.

**Figure 4:**
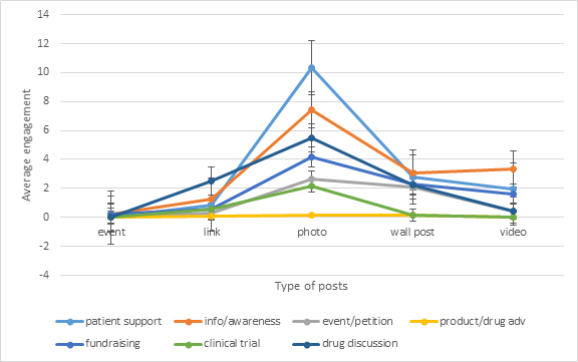
Average engagement level by type of post (event, link, photo, wall post, and video) with respect to the belonging topic with standard error.

The results show that wall posts and links are the 2 main types of post used within our dataset followed by a good portion of photos and a little percentage of videos. Wall posts are the most used type for 4 categories of content—patient support, information and awareness, event advertising and petitions, fundraising, and drug discussion—while links are the main type for 2 categories only: product and drug advertising and clinical trials and research studies. In addition, we tried to understand if the engagement is higher with respect to the type of post as shown in Figure 4. The chart clearly shows that engagement level is higher for photo posts no matters which topic.

### Results of Analysis of Variance

One of the aims of this research was to test if SNSs are valid tools to share drug information by patients. For this reason, the means of the engagement for each topic was analyzed using 1-way ANOVA. Our objective was to see if the drug discussion category received as much attention as the most well-known topics: patient support and information and awareness. In fact, according to other research, these last 2 topics seem to be recurrent topics [8,11]. For the purpose of the ANOVA, the topics have been coded as follows: drug discussion (A), information and awareness (B), event advertising and petitions (C), product and drug advertising (D), fundraising (E), clinical trials and research studies (F), patient support (G), and other (H). The results of the ANOVA in Table 5 show that *P*<.001, so we clearly reject the null hypothesis of equal engagement means for all topics, and we claim that at least one category in our topics is different from the others in terms of engagement means.

**Table 5:** Analysis of variance.

	Degrees of freedom	Sum of squares	Mean square	*F* value	Pr(>F)^a^
Topic	7	65	9.259	9.335	1.46e–11^b^
Residuals	7021	6964	0.992	—^c^	—

^a^Pr(>F): significance probability value.

^b^*P*<.001.

^c^Not applicable.

**Figure 5:**
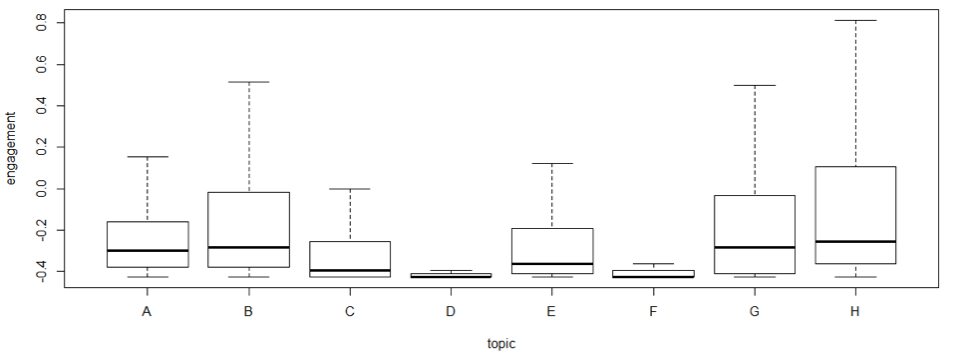
Boxplot showing engagement mean by category.

To test which categories differ, we analyzed a boxplot chart, shown in Figure 5, that shows that drug discussion (A), information and awareness (B), and patient support (G) have higher engagement means with respect to the other categories (H includes posts not belonging to any topics and so not considered in this discussion).

### Limitations

This research is based on a dataset that comes from 2 Facebook groups, and this may represent a limitation since there might be different online behaviors in other social groups. Furthermore, this study focuses only on MS groups, and we cannot argue that the results of our analysis might apply to other conditions and that we could expect the same behavior for other diseases. Another limitation of this study is represented by the fact that we don’t have demographics for the authors of the posts collected in this study. This doesn’t allow us to analyze differences based on background and demographics such as age, gender, etc.

This work has many limitations, but it represents a starting point that can be used for similar problems that attempt to structure health information. The development of dictionaries to study health-related posts can be considered one of the main potential uses of this study that can be applied to others. In fact, this methodology can be reproduced on other groups or documents that contain medical contents (with English as primary language) and express patient opinions on medical topics.

### Conclusions

SNSs have become important tools for patients and health care practitioners to share or seek information. In particular, they have often been studied in relation to chronic diseases. Other works have highlighted the use of SNSs and their level of engagement for these types of diseases. For example, Hale et al [11] showed that the engagement level for chronic diseases such as cancer and diabetes is very strong in terms of likes. Setoyama et al [9] studied the participation in online communities by patients affected by breast cancer and the related benefits. They found that there is a difference between posters and lurkers and that posters felt they received more benefits from online communities than lurkers did.

We think there is a need to understand how the information shared by patients affected by chronic diseases can be structured and used for medical research advancement. This research was a first attempt to identify the type of information shared, its structure, and its relation to the public reactions. From the initial results we were able to classify 8 different categories: patient support, information and awareness, event advertising and petitions, product and drug advertising, fundraising, clinical trials and research studies, drug discussion, and other. These findings give us a better understanding of what kind of health contents are disseminated within a community of people that hold an interest in health care and in a specific condition (eg, MS). Our results also show how content is perceived by the public. This may lead to useful applications in terms of prevention campaigns, educational programs, and therapy management. Certain information belonging to categories such as patient support, information and awareness, and drug discussion received higher attention from the public and this implies that SNSs may be used as an educational and prevention tool by increasing awareness. At the same time, patients sharing information about the treatment they are undergoing and its interaction with other medications and circumstances may represent useful insights for pharmaceutical companies or regulatory institutions to consider new scenarios and variables that were not included in their studies. As pointed out by Moorhead et al [10], SNSs are powerful tools that offer collaboration between users and provide a social interaction mechanism for many individuals. We believe that SNSs can be useful to enhance medical research and consequently health care delivery.

Our assumption was that if we were able to observe patients sharing specific drug information which then have the same level of attention as other types of posts (such as awareness, patient support, etc), we could use SNSs for gathering drug-related information. Further, the public is indeed interested in this information. We have observed this in our study and can claim that policy makers should address this phenomenon by motivating pharmaceutical companies to create SNS groups, pages, and other internet tools to provide locations where patients can publicly share the pros and cons associated with treatments they are taking.

These findings suggest that Facebook and SNSs may be valuable for disseminating health information and promoting healthy behavior by providing support and useful information that may not be available using more traditional tools.

## Abbreviations

ANOVA: analysis of variance
API: application program interface
mg: milligram
MS: multiple sclerosis
SNS: social network site
